# Low suction on digital drainage devices promptly improves post-operative air leaks following lung resection operations: a retrospective study

**DOI:** 10.1186/s13019-021-01485-z

**Published:** 2021-04-21

**Authors:** Suguru Mitsui, Shunsuke Tauchi, Takahiro Uchida, Hisashi Ohnishi, Toshio Shimokawa, Satoshi Tobe

**Affiliations:** 1 Department of Thoracic Surgery, Akashi Medical Center, 743-33 Okubocho Yagi, Akashi, Hyogo 674-0063 Japan; 2 Department of Respiratory Medicine, Akashi Medical Center, Akashi, Japan; 3 Clinical Study Center, School of Medicine, Wakayama Medical University Hospital, Wakayama, Japan

**Keywords:** Prolonged air leaks, Chest drain, Lung cancer, Low-pressure suction, Digital drainage system

## Abstract

**Background:**

We investigated the most effective suction pressure for preventing or promptly improving postoperative air leaks on digital drainage devices after lung resection.

**Methods:**

We retrospectively analyzed the postoperative data of 242 patients who were monitored with a digital drainage system after pulmonary resection in our institution between December 2017 and June 2020. We divided the patients into three groups according to the suction pressure used: A (low-pressure suction group: − 5 cm H_2_O), B (intermediate-pressure group: − 10 cm H_2_O), and C (high-pressure suction group: − 20 cm H_2_O). We evaluated the duration of air leaks, timing of chest tube replacement, the amount of postoperative air leak, volume of fluid drained before chest tube removal, and the total number of air leaks during drainage.

**Results:**

In total, 217 patients were included in this study. The duration of air leaks gradually decreased with significant difference between the groups, the highest decrease in A, the lowest decrease in C (*P* = 0.019). Timing of chest tube replacement, on the other hand, did not significantly differ between the three groups (*P* = 0.126). The number of postoperative air leaks just after surgery did not significantly differ between the three groups (*P* = 0.175), but the number of air leaks on postoperative day 1 were fewest in group A, then B, and greatest in group C (*P* = 0.033). The maximum amount of air leaks during drainage was lowest in A, then B, and highest in C (*P* = 0.036). Volume of fluid drained before chest tube removal did not significantly differ between the three groups (*P* = 0.986).

**Conclusion:**

Low-pressure suction after pulmonary resection seems to avoid or promptly improve postoperative air leaks in digital drainage devices after lung resection.

**Trial registration:**

This is a single-institution, retrospective analysis-based study of data from an electronic database. Study protocol was approved by the Akashi Medical Center Institutional Research Ethics Board (approval number: 2020–9).

## Background

Postoperative air leaks after pulmonary resection are a major factor in keeping patients in bed and in delaying early hospital discharge. Chest tubes are critical components in postoperative management of air leakage, affecting hospital stay and costs [1, 2]. Moreover, the removal of chest tubes reduces pain and improves ventilatory function in the early postoperative course [3]. Chest drainage tubes are routinely placed in the pleural space for promotion of re-expansion and for evaluation of postoperative air leaks and bleeding after pulmonary resection. Many studies have examined the optimum chest tube management for postoperative air leaks after pulmonary resection. Chest tube drainage followed by water seal could reduce the duration of air leaks and chest tube replacement after pulmonary resection [4, 5]. In recent years, with the advent of the digitally monitored thoracic system (Medela, Healthcare, Baar, Switzerland), quantitative and temporal evaluations of postoperative air leakage and pleural pressure after pulmonary resection have become possible. We hypothesized that low negative pressure suction on the chest tube after pulmonary resection could shorten the duration of air leakage and reduce the chest tube replacement time.

## Methods

This study includes the quantitative and temporal evaluation of the most effective suction pressure for prevention or early improvement of postoperative air leakage using the digitally monitored thoracic system. This is a single-institution, retrospective analysis-based study of data from an electronic database. Study protocol was approved by the Akashi Medical Center Institutional Research Ethics Board (approval number: 2020–9).

### Patient data

We collected the postoperative data of the 242 patients who were monitored in our institution with a digital drainage system (Thopaz, Medela, Inc., Baar, Switzerland) after pulmonary resection. Included were patients who had lung resection using this digital drainage system between December 2017 and June 2020. We divided the patients into three groups depending on the suction pressure used: A (low-pressure suction group, − 5 cm H_2_O, October 2019 to June 2020), B (intermediate-pressure group, − 10 cm H_2_O May 2018 to October 2019), and C (high-pressure suction group, − 20 cm H_2_O, December 2017 to May 2018). Decisions regarding a setting period of respective suction pressure were made by the attending surgeons. The suction pressure was set at − 20 cm H_2_O from the beginning. We changed the suction pressure from − 20 cm H_2_O to − 10 cm H_2_O, expecting the decrease of the complication rate since 2018 May. According to the previous report in which low suction pressure significantly shortened the duration of air leaks and chest drain replacement [6], we switched the suction pressure from − 10 cm H_2_O to − 5 cm H_2_O since October 2019. We excluded patients that required reoperation, those requiring re-drainage, those with pleurodesis after surgery, and any patients that died perioperatively. Patients treated by re-drainage or pleurodesis were excluded because they would have remained in the study for a long period of time owing to chest tube replacement time, and because their data would have had limited clinical relevance to the outcomes.

### Intervention

Patients underwent lobectomy, segmentectomy, and wedge resection for benign and neoplastic diseases. Surgery was performed by video assisted thoracoscopic surgery (VATS) and posterolateral thoracotomy. Before closing a surgical incision, a sealing test was performed. The method of sealing air leaks was managed with soft coagulation system. We also covered the stapler line and bronchial stump with polyglycolic acid sheets and fibrin glue to prevent further air leaks in patients undergoing lobectomy or segmentectomy. One chest tube (18–20 Fr) was inserted before closure of the chest. After the surgery, the digital drainage system (Thopaz) was connected. The suction pressure in each group was set according to the abovementioned group assignment immediately after the operation until removal of the chest tube. The chest tube was removed when an air leak was < 20 ml/min and there were no spikes for at least 8 h, and when the fluid drainage was < 200 ml over 24 h. In patients undergoing lobectomy or segmentectomy, chest tubes were removed after at least two postoperative days. Clinical outcomes were duration of air leaks, duration of chest tube replacement, the amount of one-day postoperative air leak, fluid volume drained before chest tube removal, and the maximum amount of air leaks during drainage. We analyzed clinical outcomes with ThopEasy software (Thopaz, Medela, Inc., Baar, Switzerland).

### Statistical analysis

Demographic characteristics of the patients are described using the mean with standard deviation (SD) for continuous variables and the frequency with proportion for categorical variables. The Kruskal-Wallis test was used to compare the continuous outcomes, and Wilcoxon test with Holm adjustment was used to compare the three groups pairwise. To assess the trends of the chest and outcomes, we performed Jonckheere-Terpstra trend tests. All statistical analyses were performed with EZR software version 1.40 (Saitama, Medical Center, Jichi Medical University, Saitama, Japan), a graphical user interface for R (The R Foundation for Statistical Computing, Vienna, Austria) [7]. A *P* value < 0.05 was considered statistically significant.

## Results

**Fig. 1:**
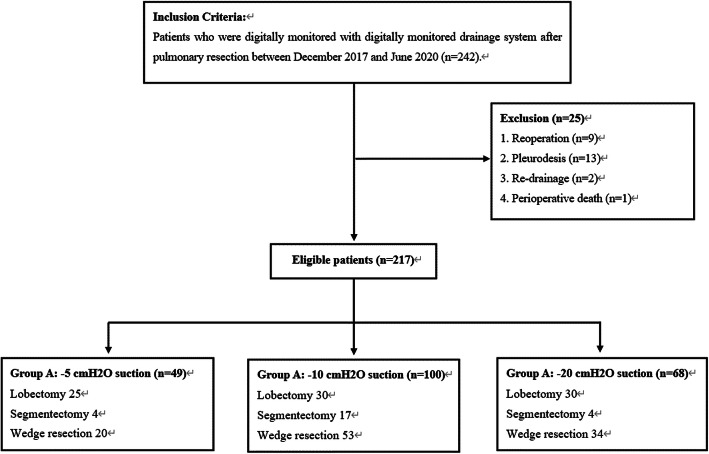
Patient algorithm for this retrospective study

We analyzed data from 242 consecutive patients who were digitally monitored by Thopaz system after undergoing pulmonary resection during the study period (Fig. 1). Patient characteristics and operative data are presented in Table 1. Of these patients, 25 met the exclusion criteria; nine patients underwent reoperation because of postoperative air leaks (*n* = 7) or bleeding (*n* = 2), thirteen patients required pleurodesis, and two patients treated with re-drainage needed additional thoracostomy because of severe subcutaneous emphysema leading to lung expansion failure after surgery. One patient died of perioperative cardiac disease.

**Table 1:** Patient characteristics and operative data

Characteristics	No. (%) (***n*** = 217)		
	Low-pressure suction (***n*** = 49) Group A	Intermediate-pressure suction (***n*** = 100) Group B	High-pressure suction (***n*** = 68) Group C
Sex (males/females)	37/12	73/27	46/22
Age (mean ± SD)	63.7 ± 19.1	54.8 ± 24.2	58.2 ± 20.9
Primary disease			
Lung Cancer	28	47	31
Metastatic Cancer	4	2	3
Pneumothorax	14	38	27
Others	3	13	7
Comorbidities			
Emphysema	8	9	7
Interstitial pneumonia	2	4	2
Diabetes mellitus	5	9	3
VATS	48 (98)	94 (94)	66 (97)
Surgical procedure			
Anatomical resection	29 (59)	47 (47)	34 (50)
Wedge resection	20 (41)	53 (53)	34 (50)

Patients comprised 156 men and 61 women with ages ranging between 14 and 94 years (median 58 years). The most common diagnosis was primary lung cancer (*n* = 106), followed by pneumothorax (*n* = 79), and lung metastases (*n* = 9). Anatomical pulmonary resection was required in 110 patients (51%), and there were 107 wedge resections.

Group A, the low-pressure suction group, comprised 49 patients (25 underwent lobectomy, 4 underwent segmentectomy, 20 wedge resection). Of these patients, three met the exclusion criteria (1 underwent reoperation, 1 required pleurodesis, 1 died). Group B, the intermediate-pressure group, comprised 100 patients (30 underwent lobectomy, 17 underwent segmentectomy, 53 wedge resection). Of these patients, sixteen met exclusion criteria (7 underwent reoperation, 7 required pleurodesis, 2 required re-drainage). Group C, the high-pressure group, comprised 68 patients (30 underwent lobectomy, 4 underwent segmentectomy, 34 wedge resection). Of these patients, six met exclusion criteria (1 underwent reoperation, 5 required pleurodesis). Patient characteristics were not distributed evenly. There were no statistically significant differences between the groups in terms of sex, age, primary disease, comorbidities, VATS, or surgical procedures. An intraoperative air leak was observed in 87 patients (40%), 22 in group A (45%), 36 in group B (36%), and 29 in group C (43%). Postoperative air leak was observed in 56 patients (26%), 8 in group A (16.3%), 24 in group B (24%), and 24 in group C (35%).

Clinical outcomes are presented in Table 2. Assessment of air leaks of patients was performed every morning. The mean duration of air leaks was 0.57 days in group A, 0.78 days in group B, and 1.13 days in group C. In the order of A, B, and C groups, duration of air leaks gradually decreased and a significant trend was observed (*P* = 0.019 by the Jonckheere-Terpstra test). In this analysis, chest tube suction pressure was significantly associated with duration of air leaks and low suction pressure promptly improved postoperative air leaks early. However, the mean duration of chest tube replacement did not significantly differ between the three groups, it was 2.12 days in group A, 2.17 days in group B, and 2.35 days in group C (*P* = 0.126 by the Jonckheere-Terpstra test). The amount of postoperative day 1 air leak was significantly the lowest in group A, then group B, and then it was highest in group C (*P* = 0.033 by the Jonckheere-Terpstra test). The amount of postoperative day 2 air leak did not significantly differ between the three groups (*P* = 0.520 by the Jonckheere-Terpstra test). The amount of postoperative air leak just after surgery did not significantly differ between the three groups (*P* = 0.175 by the Jonckheere-Terpstra test). Regarding maximum amount of air leaks during drainage, A group had the biggest decrease, followed by B group and then C group (*P* = 0.036 by the Jonckheere-Terpstra test). Mean fluid volume drained before chest tube removal was 304.5 mL in group A, 289.7 mL in group B, and 289.0 mL in group C. Fluid volume drained before chest tube removal did not significantly differ between the three groups (*P* = 0.986 by the Jonckheere-Terpstra test).

**Table 2:** Clinical outcomes

Clinical Outcome	Low Pressure Group A	Intermediate Pressure Group B	High Pressure Group C	***P*** value (difference between groups)			***P*** value (Jonckheere-Terpstra test statistics)
				A vs B	A vs C	B vs C	
Duration of air leaks, d	0.57 ± 1.60	0.78 ± 1.65	1.13 ± 1.70	0.297	**0.016**	0.055	**0.019**
Duration of chest tube replacement, d	2.12 ± 1.78	2.17 ± 1.66	2.35 ± 1.67	0.730	0.380	0.380	0.126
Postoperative air leak, mL/min	15.71 ± 48.18	6.00 ± 12.87	22.50 ± 78.92	0.454	0.089	0.110	0.060
Postoperative 1st day air leak, mL/min	7.76 ± 28.00	3.40 ± 8.31	8.53 ± 16.41	0.353	**0.035**	**0.041**	**0.033**
Postoperative 2nd day air leak, mL/min	7.59 ± 23.55	3.12 ± 8.15	8.40 ± 29.51	0.980	0.980	0.980	0.520
Maximum air leaks, mL/min	16.94 ± 49.04	7.20 ± 16.09	27.06 ± 81.77	0.507	0.059	0.059	**0.031**
Fluid volume, mL	304.5 ± 356.6	289.7 ± 295.0	289.0 ± 280.1	1.000	1.000	1.000	0.986

Postoperative air leak: Postoperative air leak immediately after surgery. Maximum air leaks: the maximum amount of air leaks during drainage. Fluid volume: Fluid volume drained before chest tube removal

Clinical outcome in patients who underwent anatomical resection are presented in Table 3. The mean duration of air leaks was 0.90 days in group A, 1.55 days in group B, and 1.94 days in group C. There was a statistical significance in decrease between the groups. (*P* = 0.010 by the Jonckheere-Terpstra test). The mean duration of chest tube replacement was 2.72 days in group A, 3.10 days in group B, and 3.15 days in group C, there was no significant difference between the three groups (*P* = 0.141 by the Jonckheere-Terpstra test). The amount of postoperative air leaks just after surgery and postoperative day 1 was greatest in group A, followed by B and then C, with statistical significance (*P* = 0.017 and 0.008 by the Jonckheere-Terpstra test). The amount of postoperative day 2 air leak did not significantly differ between the three groups (*P* = 0.389 by the Jonckheere-Terpstra test). The maximum amount of air leaks during drainage decreased the most in group A, followed by B and then C groups (*P* = 0.010 by the Jonckheere-Terpstra test). The mean fluid volume drained before chest tube removal was 424.6 mL in group A, 459.3 mL in group B, and 477.7 mL in group C. Fluid volume drained before chest tube removal did not significantly differ between the three groups (*P* = 0.288 by the Jonckheere-Terpstra test). Clinical outcomes of patients that underwent wedge resection are presented in Table 4. There were no significant differences between the three groups.

**Table 3:** Clinical outcomes (anatomical resection)

Clinical Outcome	Low Pressure Group A	Intermediate Pressure Group B	High Pressure Group C	***P*** value (difference between groups)			***P*** value (Jonckheere-Terpstra test statistics)
				A vs B	A vs C	B vs C	
Duration of air leaks, d	0.90 ± 2.00	1.55 ± 2.13	1.94 ± 1.98	0.175	**0.009**	0.194	**0.010**
Duration of chest tube replacement, d	2.72 ± 2.03	3.10 ± 2.00	3.15 ± 1.96	0.580	0.250	0.580	0.141
Postoperative air leak, mL/min	26.20 ± 60.79	8.51 ± 12.15	42.94 ± 108.36	0.467	0.061	0.061	**0.017**
Postoperative 1st day air leak, mL/min	12.76 ± 35.75	6.81 ± 11.05	15.59 ± 20.63	0.160	**0.019**	0.082	**0.008**
Postoperative 2nd day air leak, mL/min	8.46 ± 24.77	5.22 ± 10.05	12.06 ± 35.31	0.830	0.790	0.830	0.389
Maximum air leaks, mL/min	28.28 ± 61.59	8.93 ± 12.55	51.18 ± 111.02	0.647	**0.043**	**0.020**	**0.010**
Fluid volume, mL	424.6 ± 413.5	459.3 ± 349.0	477.7 ± 354.8	0.940	0.940	0.940	0.288

Postoperative air leak: Postoperative air leak immediately after surgery. Maximum air leaks: the maximum amount of air leaks during drainage. Fluid volume: Fluid volume drained before chest tube removal

**Table 4:** Clinical outcomes (wedge resection)

Clinical Outcome	Low Pressure Group A	Intermediate Pressure Group B	High Pressure Group C	***P*** value (difference between groups)			***P*** value (Jonckheere-Terpstra test statistics)
				A vs B	A vs C	B vs C	
Duration of air leaks, d	0.10 ± 0.45	0.09 ± 0.40	0.32 ± 0.77	0.940	0.390	0.220	0.363
Duration of chest tube replacement, d	1.25 ± 0.72	1.36 ± 0.52	1.56 ± 0.75	0.300	0.120	0.300	0.079
Postoperative air leak, mL/min	0.50 ± 2.24	3.77 ± 13.19	2.06 ± 6.40	1.000	1.00	1.00	0.706
Postoperative 1st day air leak, mL/min	0.50 ± 2.24	0.38 ± 1.92	1.47 ± 4.36	0.830	0.820	0.450	0.596
Postoperative 2nd day air leak, mL/min	–	–	–	–	–	–	–
Maximum air leaks, mL/min	0.50 ± 2.24	5.66 ± 18.66	2.94 ± 7.19	0.770	0.530	0.770	0.466
Fluid volume, mL	130.35 ± 123.86	169.88 ± 173.06	136.19 ± 119.56	1.000	1.00	1.000	1.000

Postoperative air leak: Postoperative air leak immediately after surgery. Maximum air leaks: the maximum amount of air leaks during drainage. Fluid volume: Fluid volume drained before chest tube removal

## Discussion

Postoperative air leaks after pulmonary resection are major factors in the recuperation period. Postoperative air leakage generally stops spontaneously after a period of few hours up to few days [4, 5, 8]. Prolonged air leaks can cause various complications, however, such as atrial fibrillation, thromboembolism, emphysema, pneumonia and respiratory failure, which lead to prolonged hospital stay and increasing costs [1, 2]. There have been various approaches to chest drain management for postoperative air leaks. Alphonso et al. and Brunelli et al. described no differences between suction groups and non-suction groups in duration of air leaks in their randomized studies of the patients that underwent pulmonary resection [9, 10]. Meanwhile, Leo et al. suggested the routine use of external suction reduces the rate of prolonged air leaks after anatomic lung resection [11], Gocyk et al. reported in their prospective randomized study that non-suction drainage is more effective than suction drainage regarding drainage volume, drainage duration, and the rate of prolonged air leak [5]. In recent meta-analysis of randomized controlled trials on the effect of the addition of suction to water-seals on the postoperative outcomes in patients undergoing pulmonary resection, there were no significant differences between the suction and non-suction groups concerning duration of air leaks, hospitalization, or in occurrence of prolonged air leaks [12]. Where there is concern about residual or increasing pneumothorax, it was concluded that the addition of suction should be carefully, selectively considered. Several limitations were suggested, however, including heterogeneity among patients included in the study, lack of standard procedures for chest tube management, and insufficient sample size. The optimum postoperative suction pressure remains controversial.

Evaluation of the amount of air leak by traditional closed chest tube drainage system has been difficult. Recently, however, with the advent of the digitally monitored drainage system, it became possible to perform quantitative and temporal evaluations of postoperative air leaks and pleural pressure after pulmonary resection. Additionally, digitally monitored drainage system is expected to be a useful predictor of postoperative prolonged air leaks [13]. There are advantages of evaluation of the postoperative course after pulmonary resection by digitally monitored drainage system. Miller et al. reported a single-center study of 108 patients that underwent VATS lobectomy or segmentectomy [14]. Digitally monitored drainage system was shown to reduce the duration of air leaks, the need for chest tube replacement, hospitalization and the rate of complications. Moreover, there was significant reported reduction in the duration of air leaks, the need for chest tube replacement, and hospitalization compared with traditional closed chest tubes [15]. Even in meta-analysis of randomized controlled trials, the effect of the digitally monitored drainage system compared with the traditional closed chest tube in patients undergoing pulmonary surgery was reduction in duration of air leaks and the need for chest tube placement, and hospitalization [16]. In another recent meta-analysis, the digital drainage system was said to benefit patients in attaining faster recovery and higher quality of life as well as reduction of the risk of postoperative complications [17].

The main strength of our study is that the effect of chest tube suction pressure on the postoperative air leaks was quantitatively evaluated. Duration of air leaks had clear tendency to be significantly shortened with decreased suction pressure. Holbek et al. reported similar findings in a prospective randomized study [6]. Their 228 patients undergoing VATS lobectomy were allocated to either − 2 cmH2O or -10cmH2O suction drainage on digital pleural drainage device after surgery. They concluded that low suction pressure significantly shortened the duration of air leaks and chest drain replacement. In the current study, however, duration of chest tube replacement did not significantly differ between the three groups, perhaps due to the chest tube removal after at least two postoperative days in patients undergoing lobectomy or segmentectomy. In addition, low-pressure suction tends to make the amount of air leak fall just after surgery and on postoperative 1 day. There were no significant differences on postoperative day 2 in our analysis of anatomical pulmonary resection. In conclusion, we suggest that low-pressure suction, at least on postoperative 1 day, may help early improvement of air leakage in patients whose air leakage can be expected to stop spontaneously. Gentle handling of the lungs by low-suction pressure probably allows for early improvement of damage of lung parenchyma and prevents new tears in lung parenchyma. There was no significant difference in fluid volume drained before chest tube removal between the three levels of suction pressure. We believe high-pressure suction drainage is unnecessary when postoperative pleural effusions and hemorrhage are considered. The difference across all categories between low-suction pressure group (group A) and intermediate-suction pressure group (group B) is unclear, but there is a good possibility that more accumulation was significant due to differences in the number of cases. Clinical outcomes of patients who underwent wedge resection did not significantly differ between the three groups. Given the lower numbers of postoperative air leakage among the patients who underwent wedge resection, this may be attributable to a lack of statistic power.

This study has several limitations. As a retrospective study, it was performed without the randomization of patient selection and in a single center. Patient characteristics did not have significant difference in all categories between three groups, but we cannot not deny some sample bias could distort our result because it was not randomized. In addition, the number of excluded cases increased to 25 cases because the next treatment criteria for postoperative air leakage was undecided. In some cases, pleurodesis and surgery were performed early after surgery, and in some cases second treatments were performed after 1 week, so we had no choice but to exclude all cases of second treatment for postoperative air leakage and bleeding.

## Conclusion

Low suction pressure (− 5 cm H_2_O) shortened the duration of air leaks after pulmonary resection compared with groups that had higher suction pressure (− 10 cm H_2_O and − 20 cm H_2_O), although fluid volume drained before chest tube removal did not significantly differ between the three groups. Low-pressure suction after pulmonary resection could prevent or promptly improve postoperative air leakage after pulmonary resection.

## Abbreviation

VATS: Video assisted thoracoscopic surgery

## References

[1] Okereke I, Murthy SC, Alster JM, Blackstone EH, Rice TW (2005). Characterization and importance of air leak after lobectomy. Ann Thorac Surg.

[2] Irshad K, Feldman LS, Chu VF (2002). Causes of increased length of hospitalization on a general thoracic surgery service: a prospective observational study. Can J Surg.

[3] Refai M, Brunelli A, Salati M, Xiumè F, Pompili C, Sabbatini A (2012). The impact of chest tube removal on pain and pulmonary function after pulmonary resection. Eur J Cardiothorac Surg.

[4] Marshall MB, Deeb ME, Bleier JI, Kucharczuk JC, Friedberg JS, Kaiser LR (2002). Suction vs water seal after pulmonary resection: a randomized prospective study. Chest..

[5] Gocyk W, Kużdżał J, Włodarczyk J, Grochowski Z, Gil T, Warmus J, Kocoń P, Talar P, Obarski P, Trybalski Ł (2016). Comparison of suction versus nonsuction drainage after lung resections: a prospective randomized trial. Ann Thorac Surg.

[6] Holbek BL, Christensen M, Hansen HJ, Kehlet H, Peterson RH (2019). The effect of low suction on digital drainage devices after lobectomy using video-assisted thoracoscopic surgery: a randomized controlled trial. Eur J Cardiothorac Surg.

[7] Kanda Y (2013). Investigation of the freely-available easy-to-use software “EZR” (easy R) for medical statistics. Bone Marrow Transplant.

[8] Mueller MR, Marzluf BA (2014). The anticipation and management of air leaks and residual spaces post lung resection. J Thorac Dis.

[9] Alphonso N, Tan C, Utley M, Cameron R, Dussek J, Lang-lazdunski L (2005). A prospective randomized controlled trial of suction versus non-suction to the under-water seal drains following lung resection. Eur J Cardiothorac Surg.

[10] Brunelli A, Monteverde M, Borri A, Salati M, Marasco R, Refai M (2004). Comparison of water seal and suction after pulmonary lobectomy: a prospective, randomized trial. Ann Thorac Surg.

[11] Leo F, Duranti L, Girelli L, Furia S, Billè A, Garofalo G, Scanagatta P, Giovannetti R, Pastorino U (2013). Does external pleural suction reduce Prolinged air leak after lung resection? Results from the AirINTrial after 500 randomized cases. Ann Thorac Surg.

[12] Zhou J, Chen N, Hai Y, Lyu M, Wang Z, Gao Y, Pang L, Liao H, Liu L (2019). External suction versus simple water-seal on chest drainage following pulmonary surgery: an updated meta-analysis. Interact Cardiovasc Thorac Surg.

[13] Goto M, Aokage K, Sekihara K, Miyoshi T, Tane K, Yokoi K, Tsuboi M (2019). Prediction of prolonged air leak after lung resection using continuous log data of flow by digital drainage system. Gen Thorac Cardiovasc Surg.

[14] Miller DL, Helms GA, Mayfield WR (2016). Digital drainage system reduces hospitalization after video-assisted thoracoscopic surgery lung resection. Ann Thorac Surg.

[15] Takamochi K, Imashimizu K, Fukui M, Maeyashiki T, Suzuki M, Ueda T, Matsuzawa H, Hirayama S, Matsunaga T, Oh S, Suzuki K (2017). Utility of objective chest tube management after pulmonary resection using a digital drainage system. Ann Thorac Surg.

[16] Zhou J, Lyu M, Chen N, Wang Z, Hai Y, Hao J, Liu L (2018). Digital chest drainage is better than traditional chest drainage following pulmonary surgery: a met-analysis. Eur J Cardiothorac Surg.

[17] Wang H, Hu W, Ma L, Zhang Y (2019). Digital chest drainage system versus traditional chest drainage system after pulmonary resection: a systematic review and meta-analysis. J Cardiothorac Surg.

